# Why do dentists refrain from intervention in cases of persistent asymptomatic apical periodontitis in root canal filled teeth? An interview study among general dental practitioners

**DOI:** 10.1111/iej.14158

**Published:** 2024-10-23

**Authors:** Maria Granevik Lindström, Helena Fransson, Victoria S. Dawson, Thomas Kvist, EndoReCo, L. Bjørndal, V.S. Dawson, H. Fransson, F. Frisk, P. Jonasson, T. Kvist, M. Markvart, M. Pigg, E. Wigsten, Eva Wolf

**Affiliations:** ^1^ Department of Endodontics, Faculty of Odontology Malmö University Malmö Sweden; ^2^ Specialist Clinic Kaniken Public Dental Health Service Uppsala Sweden; ^3^ Department of Endodontology, Institute of Odontology, Sahlgrenska Academy University of Gothenburg Gothenburg Sweden; ^4^ Endodontic Research Collaboration in Scandinavia

**Keywords:** [clinical] decision‐making, endodontics, periapical periodontitis, qualitative research, retreatment, tooth extraction

## Abstract

**Aim:**

To explore the context in which general dental practitioners (GDPs) decide to refrain from further treatment, that is, ortho‐ or retrograde retreatment or extraction of a root canal filled tooth with persistent asymptomatic apical periodontitis (PAAP).

**Methodology:**

Fifteen GDPs were strategically selected for in‐depth interviews. The informants were encouraged to describe in their own words and in as much detail as possible, the three most recent patient cases of PAAP of a root canal filled tooth, in which they decided to refrain from further treatment. The interviews were recorded digitally and transcribed *verbatim*. The collected material was analysed according to Qualitative Content Analysis with an inductive approach.

**Results:**

A pattern of varying degrees of uncertainty associated with the decision process was identified. The motives to refrain intervention had great diversity. The result from analysis of the qualitative data was formulated in an overall theme ‘Between doubt and certainty in a complex clinical context’ covering the latent content. The first main category covering the manifest content was ‘The continuum of confidence’ with three subcategories ‘Experienced uncertainty’, ‘Reluctant approval’ and ‘At ease with refraining’ illustrating the feelings and attitudes experienced by the informants. The second category was ‘In support of acceptance’ with three subcategories ‘Patient's autonomy, risks and cost‐benefits’, ‘Emotional aspects’ and ‘Relieving measures’ representing the reasons for refraining from intervention.

**Conclusions:**

The decision to refrain from further treatment, that is, ortho‐ or retrograde retreatment or extraction of a root canal filled tooth with PAAP was made with some measure of confidence, combined with compensatory strategies to support the decision, taking into account not only values beneficial to the patient and awareness of limited external resources, but also factors related to the informants' personal preferences, convenience, concerns, ambition and emotions.

## INTRODUCTION

Epidemiological surveys imply that asymptomatic apical periodontitis (AP) is prevalent in many root canal filled teeth (RCFT) (Jakovljevic et al., 2020; Pak et al., 2012; Silnovic et al., 2022). These are often left without further treatment, substantiated by the fact that general dental practitioners (GDPs) rarely perform retreatments (Bjørndal et al., 2006; Dawson et al., 2017; Hommez et al., 2003; Olsson et al., 2024; Wigsten et al., 2019). Though RCFT are more frequently extracted than non‐RCFT (Eckerbom et al., 2007), the reasons for extraction are more likely to be fractures and caries than presence of AP (Chen et al., 2008; Ng et al., 2010).

In this context, it is of interest to investigate the GDP's decision‐making process of RCFT with persistent asymptomatic AP (PAAP). Previous research has used surveys, investigating various aspects of the decision‐maker as well how certain clinical factors influence the decisions (Al‐Ali et al., 2005; Alani et al., 2011; Burns et al., 2018; Kvist et al., 1994; Lee et al., 2020; Liew et al., 2022; Mota de Almeida et al., 2016; Olsson et al., 2022; Rawski et al., 2003; Taha et al., 2019; Wenteler et al., 2015). The abundance of factors involved, and the complexity of the process have made a concise descriptive theory of the decision making of AP in RCFT from these studies hard to get hold of (Kvist & Hofmann, 2023).

A qualitative research approach to complex decision problems in health care can offer new insights into the processes when analysis of quantitative data shown elusive to provide a profound understanding (Pope & Mays, 2020). An interview study of dentists in a hospital setting disclosed several factors which influenced the management of authentic patients presenting an RCFT with asymptomatic AP, including the size of the lesion, any medical immunosuppression, and the aesthetic and functional importance of the tooth. Furthermore, the influence of colleagues and physicians' opinions were factors that determined the choice of either retreatment/extraction or refraining from treatment in this limited patient category facing critical medical treatment (Olsson et al., 2023). To our knowledge there are no published studies using qualitative method of exploring how GDPs reason when they decide to refrain from treatment of PAAP in an RCFT.

The aim of this study was to explore, with a method for analysis of qualitative data, the reasoning in context among GDPs who decide to refrain from further treatment, that is, ortho‐ or retrograde retreatment or extraction of an RCFT with PAAP.

## MATERIALS AND METHODS

Data were collected from in‐depth interviews with GDPs, that is, the informants, who were asked to describe their experiences, perceptions, thoughts and feelings (Moser & Korstjens, 2018) in the process of reaching a decision not to treat an RCFT with PAAP. The data were analysed according to Qualitative Content Analysis with an inductive approach (Graneheim et al., 2017; Graneheim & Lundman, 2004).

### Preunderstanding

All the authors are specialists in endodontics, with several years of clinical practice. First author is a senior consultant in the Public Dental Health Service but has no previous experience of qualitative data analysis. Co‐authors are associate or assistant professors. All have experience of qualitative data analysis. The authors' preunderstanding was the assumption that GDPs are confident of their decision not to treat an RCFT with PAAP, based on their clinical experience that exacerbation of such teeth is rare.

### Context

Sweden, with a population of some 10 million, has 10 cities with more than 100 000 inhabitants. Dental education to become GDP is conducted at four different universities. Little more than half of Sweden's 6000 GDPs work within Public Dental Health Service, very few at the universities and the remainder as private practitioners (Tandvårds‐ och läkemedelsförmånsverket, 2023). Approximately 90 endodontists work in either public or private sector and serve under the same national insurance and reimbursement system as GDPs (Socialstyrelsen, 2024).

### Informants

**FIGURE 1:**
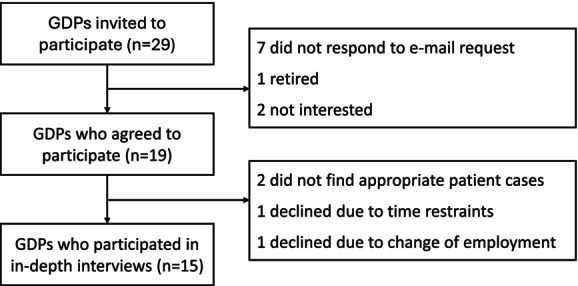
Flow‐chart of recruitment of participants, reasons for not participating and final number of participants. GDPs, general dental practitioners.

Informants was recruited based on strict inclusion criteria: personal experience of deciding not to treat RCFT with PAAP and fluency in spoken Swedish. Informants were selected strategically in order to ensure good heterogeneity among the informants and their patient clientele, exposing potentially different perspectives (Table 1) (Moser & Korstjens, 2018; Polit & Beck, 2017). The first author approached 29 potential participants practicing in Sweden via personal contact, phone or email, resulting in 15 informants (Figure 1).

**TABLE 1:** Characteristics of the informants, that is, general dental practitioners (GDP) (*n* = 15).

Mean age	49 (31–63)^a^ years
Sex	8 men
	7 women
Dental degree from	
Göteborg	1
Malmö	3
Stockholm	6
Umeå	4
European capital city	1
Mean years of practice	22 (4–38)^a^ years
≤5 years	2
6–15 years	3
16–25 years	4
26–38 years	6
Employment/type of practice	
Solo private practice 1 GDP	2
Group private practice >1 GDP	4
Public dental health service >1 GDP	9
Geographical location^b^	
Rural area, city <10 000 inhabitants	3
Minor city 10 000–100 000 inhabitants	8
Larger city >100 000 inhabitants	4
Proximity to endodontic specialist care	
The same city	6
From one to several hours travel distance	9

^a^ Distribution.

^b^ Statistics Sweden (www.scb.se).

### Data collection

The first author conducted two pilot interviews and received feedback on interview technique and appropriate follow‐up questions from the last author. These interviews were not included in the study. The first author then conducted separate in‐depth, face‐to‐face interviews (Moser & Korstjens, 2018; Polit & Beck, 2017) with the 15 strategically selected informants. Each informant was invited to narrate freely, describing the three most recent occasions on which a patient presented with PAAP in an RCFT and the informant made a decision not to treat the tooth. All interviews started with an introductory open‐ended question ‘Tell me about the last case you had with PAAP in an RCFT where you decided to refrain from retreatment of the root filling’. Other supplementary questions in the interviews could be ‘How did this (fact, problem, etc.) matter in this case?’ or ‘Could you elaborate on that thought?’ intended to deepen the informants' narrative, all with the purpose to follow the informant in his/her narration. No question guide with mandatory predetermined thematic questions were used. All interviews were held at the informant's workplace, or at another location chosen by the informant. The conversation was recorded digitally and sent for professional *verbatim* transcription. The interviews, comprising a plethora of patient cases (Table 2), were 36–90 min long and were conducted from October 2017 through August 2020.

**TABLE 2:** Overview of the cases assessed and presented by the informants at the interviews.

Types of cases presented by informants	Children to elderly of both sexes Seeking dental care on a regular or irregular basis or were patients new to the dentist Healthy, essentially healthy or with varying levels of infirmity (physical, psychological, cognitive) Varying dental care needs (none, some, extensive) Different personal economic resources, subject to different economic refund systems, or free dental care Interested or not interested in their own oral health Swedes, Europeans, Non‐Europeans
The informants discovered PAAP	At an assessment of the outcome of endodontic treatment As an additional finding At an examination of new patients including a complete radiographic examination
Informants assessed the teeth having	Root canal fillings of different quality Apical periodontitis assessed on radiographs as increased, decreased, unchanged or unknown development With or without signs of infection other than periapical radiolucency Retreatment or no previous retreatment With or without other dental diseases such as caries, marginal periodontitis, fractures, etc. Direct or indirect restorations of varying quality, in combination with or without a post and core

Abbreviation: PAAP, persistent asymptomatic apical periodontitis.

### Text preparation and analysis

**FIGURE 2:**
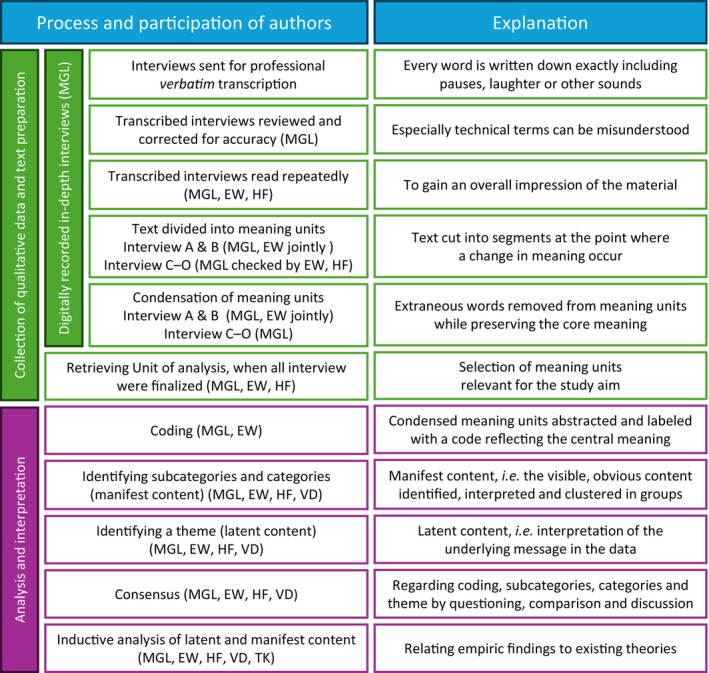
Flow‐chart of text preparation and analysis of qualitative data, according to Qualitative Content Analysis (Graneheim et al., 2017; Graneheim & Lundman, 2004), specifying the authors' contributions.

The data were processed and analysed (Figure 2) according to the method of Qualitative Content Analysis (Graneheim et al., 2017; Graneheim & Lundman, 2004). Condensed meaning units relevant for the aim of the study was abstracted and labelled with a code (Table 3) allowing formulation of categories, subcategories and a theme. The informants were not invited to comment on the transcriptions or provide feedback on the findings.

**TABLE 3:** Examples of the process of condensing and coding the meaning units.

Data source	Meaning unit	Condensed meaning unit	Code
A_04	‘… … So it was really for that reason. Ok, now I *have made a note of it* and that we need to keep an eye on it. And have also informed the patient that this happens now and again and …’	I *have made a note*, informed the patient, we keep an eye on it	Information and follow‐up plan
F_39	‘Yes. No, so … … … she is really just worried in general that it will fall apart completely and … … … And there is in fact more than a little risk of that …, that that's what will happen on the left side of the maxilla … … … … … … So I really think that trying to treat the apex of the lateral would be a bit like painting over the rust on a car’	She is worried it will fall apart completely, more than little risk on left side maxilla. I think treat apex of lateral would be like painting over rust on car	Pragmatic—Other issues are given priority

The Standards for Reporting Qualitative Research checklist was completed (O'Brien et al., 2014).

### Ethical considerations

The Regional Ethical Review Board, Lund University, Lund, Sweden, determined that the study did not require a permit according to Swedish law, but issued an advisory statement approving the study, Dnr 2017/536. Informed consent was obtained from the participants.

## RESULTS

**FIGURE 3:**
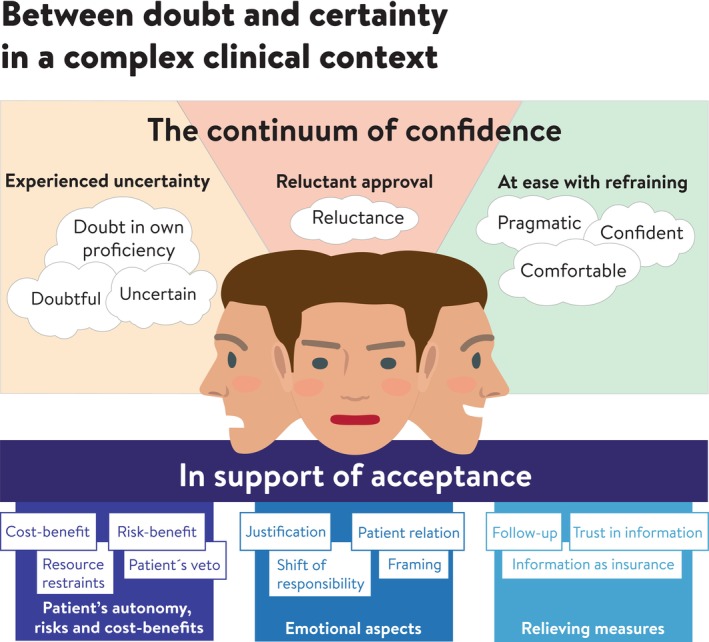
Illustration of the results of the qualitative content analysis presenting the theme, categories, subcategories and examples of codes from in‐depth interviews. The interviews were conducted with 15 general dental practitioners and dealt with patient cases of persistent asymptomatic apical periodontitis of a root canal filled tooth, in which the dentist had decided to refrain from further treatment.

The overall theme identified was ‘Between doubt and certainty in a complex clinical context’. The meaning units and codes disclosed multiple issues motivated in various ways by the informants in their decision to refrain from treatment of PAAP in an RCFT in patients in general dentistry. The codes were organized into two categories, each comprising three subcategories (Figure 3).

### The continuum of confidence (category)

This category encompasses the feelings and attitudes experienced by the informants about factors in the context. A decision not to treat an RCFT with PAAP was sometimes made with hesitation or reluctance, but sometimes with confidence that the risk of leaving the condition untreated was almost negligible.

#### Experienced uncertainty (subcategory)

The informants described a variety of situations connected to diagnostics, treatment, prognosis or consequences on general health, where they experienced uncertainty.

Concern was expressed over aspects of the diagnostic process and determination of periapical status. In all cases the RCFT had a radiolucency at the apex, but its significance was unclear.

The x‐ray doesn't really look quite ok, there is something there and we don't know what it is. Is it an infection or is there healing going on or is it going to… (Informant L)

If earlier radiographs were unavailable, uncertainty about the lesion's development reduced confidence in diagnosing PAAP, even with a clear radiolucency and long‐ago root canal treatment. Referral for cone beam computed tomography was suggested to reduce uncertainty by providing more information about the periapical status.

There could be doubt about a tooth's overall condition but also regarding successfully accomplishing a retreatment and achieving a better technical result, especially if the existing root canal filling was the informant's own work.

And what's to say that it will be better if you redo it, if you didn't succeed the first time? When all the conditions were favourable. (Informant M)

Informants were ambivalent about PAAP impact on a patient's general health and unsure about the available scientific evidence.

#### Reluctant approval (subcategory)

This subcategory entails situations where informants reluctantly were accepting a patient's refusal of intervention.

The tooth could have obvious clinical signs of infection and inflammation, such as a fistula and yet the patient would not agree to intervention, which was against the informant's advice.

But this was in fact…This was very much about the patient's wishes here, I have to say. I had in any case been thinking: No, but now…, let's extract this tooth […] But …in this case the patient persuaded me… (Informant H)

Informants felt unwilling to accept the patient's request when they suspected that refraining from intervention would risk deterioration of the situation.

So, if you wait a few years and this gets worse, and then the patient says: “yes, but now I want you to retreat this”, then…, then it's, then all of a sudden it's a much bigger infection that has to heal. Ehh… So that …, then the prognosis for… succeeding with the tooth is worse. Ehh…, and for me to succeed with my treatment. (Informant K)

The informants were frustrated and concerned that an exacerbation might occur at an inconvenient time for both parties. Furthermore, informants were bothered by the patient's veto in cases of concomitant systemic diseases. Repeated patient refusals evoked feelings of unease or disappointment, as informants felt responsible for overlooking infections. Informants struggled with patient comprehension and motivation for intervention, leading to frustration over patient's lack of disease awareness, sometimes in combination with cultural clashes. Being unable to motivate the patient could in fact evoke feelings of guilt and self‐reproach.

At first I just feel like: “Shit, what a bad dentist I am, I can't get through to the patient with my message, and I know it is perhaps the right advice for the patient.” But then one tries to do one's best. And some patients need time to think about things and some … understand afterwards. (Informant O)

#### At ease with refraining (subcategory)

Informants refrained from treatment with the pragmatic view that ‘if it isn't broken, don't fix it’, combined with their professional experience that an RCFT with PAAP represented a low risk of serious consequences.

If the patient was in good general health, the informant was more comfortable with the decision to refrain from treatment. On the other hand, informants argued that a patient who was too sick or frail should not undergo any intervention.

One can die of dementia. Because one function after another fails. So that… And… Now I didn't in fact write in the patient's record “the patient is going to die soon, so I won't undertake any treatment”. But…, but one can't help thinking that she… She is in fact a seriously ill person… With a minor…, a minor… *little laugh*… apical periodontitis. (Informant H)

Youth was considered a motive for treatment, while old age was seen as an argument in favour for refraining from intervention.

She is relatively lively. Quite clear in the head and seems very healthy. It isn't bothering her. […] She is 90 years old. I'd say it's alright to leave it like that. (Informant G)

Several cases involved teeth that were candidates for extraction due to issues other than the PAAP, such as a suspected vertical root fracture, caries, severe marginal periodontitis, extensive loss of tooth substance or complications such as fractured files or perforations. The patient could have vetoed extraction, but sometimes the informant just postponed intervention noting the tooth was still functioning.

One could have given more thought to extracting the tooth. But in his case, he is very dependent on it, in order to…, in order to bite, quite simply. And as I said, I think there is this fractured file here at…, which means that it…, so there hasn't like been a question of anything more than putting in a screw post and doing an amalgam crown there. (Informant N)

The informant could regret taking the radiograph which disclosed the PAAP: complicating the situation with unwanted information. In other cases, RCFT with PAAP had been stable for years or decades. Informants were confident that the lesion was unlikely to harm the patient and found retreatment unnecessary.

And there is no risk with this as it is. If anything happens, one can intervene then. If nothing happens, what does it matter… if it looks the way it does? (Informant G)

To refrain from intervention could be a pragmatic way of evading complicated procedures not only for the patient but also for the informant. Informants felt assured that the prognosis for retreatment was doubtful and leaving the tooth untouched was the best course of action. It could also be a way of avoiding or postponing a high‐risk treatment procedure.

### In support of acceptance (category)

This category comprises the motives the informants presented, to explain why they had decided to refrain from treating the RCFT. To compensate other arrangements then had to be made.

#### Patient's autonomy, risks and cost‐benefits (subcategory)

Informants balanced venture versus utility and found no justification, on an individual or societal level, to recommend intervention.

Informants told the patient about the tooth's status and treatment options, but the patient could decline intervention. The patients' sovereignty overruled any arguments from the informant.

But the patient has the last word. One can't do anything if they don't agree. That's the way it is … (Informant O)

Informants avoided interventions that could risk deteriorating the patients' oral status due to complications like perforation, fractures and loss of dentine, jeopardizing tooth survival. They were concerned about creating problems, such as exacerbations, for currently problem‐free patients. They also considered potential adverse effects on general health.

And just when one is able to present the advantages of taking the tooth out, in relation to like what it could have for negative consequences, what it will mean with everything, the added stress of an operation and like the local anesthesia and maybe sedative medication and maybe he'll need to have a general anesthetic with all those risks, like, if it comes to that….Ehh… …And the risk of like postoperative infections and things like that, which …., which we can… … …aren't so common, like, but they still happen and…, and … … they can like be serious too. (Informant I)

Costs versus benefits on the patients' regard were considered, including fees, income loss and patient inconvenience. Resource limitations, such as time pressures and societal costs, also influenced decisions to refrain from intervention.

#### Emotional aspects (subcategory)

This subcategory comprises the challenge of dealing with sometimes conflicting wishes and demands, both internal and external.

To relieve the burden of making difficult decisions alone, informants sought support in the opinion of colleagues.

So part of you thinks that there was a risk in going ahead with treatment. […] Then you think that there is also a risk in leaving it alone too. If it is in fact an active infection… … in a patient on bisphosphonates. …, […] this is when I have in fact consulted… *laugh* … an endodontist. Haven't made the decision completely independently. (Informant H)

To shift liability for the decision, the informant referred to previous colleagues' decisions. Another way was framing information, hoping the patient would decline treatment, thus relieve the informant of sole responsibility.

How I presented this discussion for the patient… In this case he was very receptive to what I said. … … And therefore…, so at first, so…, so he decided to take out the tooth. Because at this stage I might have, have pointed out that this was in fact something that needed to be treated. … Whereas the second time I talked to the patient perhaps I tried to point out that perhaps… …Yes. Perhaps it isn't absolutely necessary to do something about it right now […] … I have reflected about this afterwards…, that really, I was quite happy for the patient to make the decision (Informant L)

Orthograde retreatment was seen as time‐consuming and technically difficult, especially engaging previously untreated parts of the canal system. Informants could feel a lack of support from assisting personnel and financial coverage concerns counteracting a decision for retreatment.

Yes, but it takes a lot of patience to like have to concentrate for so long…. You have to keep very calm … And watch what you are doing and at the same time keep a close eye on the clock, and like know … make decisions: Shall…, I think I'm going to find that canal soon. But I… I'm due to see my next patient in ten minutes. Shall I keep looking for the canal or close up, and then book a further appointment? And that will blow the budget …to bits. So there are many many factors to be considered … … ehh …, all at the same time. (Informant M)

Refraining from any intervention, was sometimes the result of the informants' ‘Laissez fair’‐attitude.

And quite a lot… things that I think…, …, I haven't got a firm plan for it, just: We'll see what happens. … … And that no…, no subjective symptoms and like no…., no rush to do anything. … […] I don't know, I'm also a rather lazy type. Then … I don't know whether that's got anything to do with it, that I …, … that I'd rather keep my hands behind my back than blunder in…, … (Informant F)

Poor patient cooperation could justify refraining from intervention, as it made treatment difficult and demanding for both patient and informant.

To add to his dental fear …., scared that …Yes. “the dentist did something and it fell apart and there was blood and all, so it ended up as an extraction”, so … so it probably won't help him, …. … … …If we…, if we consider that he…, has very grave dental fear. He can't manage without Midazolam or nitrous oxide, but it's almost as if…., Yes, he looks at us as if…, as if we are murderers…, …every time it …, he comes in. (Informant B)

Informants were eager not to jeopardize the patient's confidence and trust, worrying about their professional reputation if the patient was dissatisfied with the outcome.

And most of all, the patient's trust, eh. That it…, it doesn't matter that you have done everything according to the rules. If the patient's impression is “No, but this …, it didn't feel as if I need … that it was necessary.” And especially if there is a complication, then it doesn't help at all […] that you have purely, how should I say, medically, legally, in every way … you are in the clear, eh, but it doesn't matter. Because the …, the patient can be like absolutely furious about this, eh? And …, and … yes, willingly makes his displeasure known … (Informant A)

Anticipating a negative reaction from the patient if a colleague's previous treatment had failed discouraged informants from recommending retreatment.

It also feels like… Sometimes it can feel as if … … … … Ehh… …It can also feel a little sensitive to start a complicated treatment on …, on something like this…It seems a bit as if one is taking the blame for …, because he has like been wrongly treated before. (Informant I)

#### Relieving measures (subcategory)

To gain control over the situation, the informants used a strategy of ‘information and follow‐up’, regardless of the reasons for refraining from further treatment.

A watchful waiting approach could be an active choice or a response to a patient's veto. Recorded notes could serve as insurance for the informant in case of future complaints.

It's not as if one lets it go or ignores it or doesn't say anything…, but this is… included in the notes and the patient…Yet it is always the case that the patient in fact forgets what you tell them, eh. But there is still evidence that I have …, I have informed the patient about this and…, and explained and demonstrated too. On…, on…, on the pictures (X‐ray) there. (Informant A)

When treatment was postponed, informants felt relieved instructing the patient to be observant about possible symptoms, putting the onus on the patient to seek contact if necessary. Scheduling follow‐ups provided a back‐up plan, allowing the informant to defer treatment and monitor the tooth for changes over time.

So you don't let it go, instead you keep a check on it […] And he goes to the dental hygienist every four months and I see him once a year. I mean, if there was a fistula or a deep pocket or any swelling then she will also be checking. So it will in fact…., it will be checked often. Mm (Informant D)

## DISCUSSION

This study disclosed GDPs' various degree of uncertainty and reluctance in management of patients presenting with PAAP in RCFT. This was unmistakably related to the complex clinical context, with factors such as patient's autonomy, risk assessment and costs and benefits favouring either an intervention or refraining. Besides the dentist's own clinical skills it was also clearly demonstrated that factors related to his or her own preferences and characteristics, comprising relational and emotional aspects, as well as convenience, were involved in the decision‐making process.

### Methodological considerations

We argue that to reach to an understanding of a recurrent complex clinical situation, and to explore factors of influence, collection of qualitative data is an appropriate scientific method (Pope & Mays, 2020). To analyse data a corresponding scientific method, Qualitative Content Analysis, was chosen (Graneheim & Lundman, 2004). Using in‐depth interviews and mainly open‐ended questions facilitated for the interviewer to avoid her own preconceptions and risk of directing the informant during the interview (Pope & Mays, 2020). However, the interviewer was an endodontist and the informants might have felt that their clinical decisions were being judged. On the other hand, the interviewer, with clinical experience and understanding of the clinical reality, was able to contribute insightful follow‐up questions. Conducting the interviews at a location chosen by the informant was intended to signal a shift in power in favour of the informant (Pope & Mays, 2020).

The informants' varied background and clinical experience, including the wide range of clinical cases presented during the interviews, favoured broad access to information and therefore also the possibility of saturation (Boddy, 2016). In hindsight it could be seen that all subcategories were represented after three interviews, and all types of codes after six interviews.

Regarding transferability of the results, there are several aspects to consider. A limitation of this study is that the prerequisites of the national insurance system cannot readily be projected to other contexts. Regarding access to an endodontist, all informants had the possibility to refer patients to a specialist clinic, at different geographical distances, yet easy access did not seem to affect the decision‐making in a clear‐cut way. Another issue is the strong influence of the patient's wishes resulting in discomfort and reluctance among the informants. If our findings are applicable in other countries and settings must be further evaluated in future studies.

No attempt was made to analyse the results based on demographical differences among the informants. All informants expressed a pragmatic attitude related to one or more patient cases and the choice to refrain intervention. Thus the confidence expressed by the informants was in accordance with our preunderstanding. Their experience that PAAP in RCFT follows a long uneventful course is supported by the findings of Yu et al. (2012) as well as Jonsson Sjögren et al. (2019), Petersson et al. (2016) and Van Nieuwenhuysen et al. (1994).

In order to strengthen the credibility of the study, investigator triangulation was used (Korstjens & Moser, 2018). Hence the coding was done separately by two authors and thereafter compared and agreed on after questioning, comparison and discussion, and likewise identifying categories, subcategories and the theme (Figure 2).

### Discussion of results

Previous studies on this topic have been based almost exclusively on hypothetical simulated case scenarios (Al‐Ali et al., 2005; Alani et al., 2011; Burns et al., 2018; Kvist et al., 1994; Lee et al., 2020; Liew et al., 2022; Mota de Almeida et al., 2016; Olsson et al., 2022; Rawski et al., 2003; Taha et al., 2019; Wenteler et al., 2015). However, doubts have been raised about studies describing or predicting physicians' actual behaviour on the basis of written case simulations (Jones et al., 1990). The present study reveals that the clinical condition of the tooth in question was only one of a number of factors considered in making the decision and that in several cases other factors were decisive. The patterns emerging in this study could be summarized in two parts: first – the degree of confidence with which the decision was made and second – how the decision was justified and facilitated.

Uncertainties are of different kinds and many of them appear in the context of decision‐making of RCFT with PAAP and challenges the dentists' confidence when deciding not to intervene (Kvist & Hofmann, 2023; Olsson et al., 2023; Pigg et al., 2022). To reduce certain types of uncertainty, a strategy of seeking more information can be used (Pigg et al., 2022). It has been suggested that reflection can diminish uncertainty such as when solutions to problems might be found (Brodén, 2022). To find support in the decision‐making process and reflect on the problem, the informants sought second opinions among colleagues. Scheduled sessions for discussion of patient cases can promote reflection (Pigg et al., 2022) and provide the opportunity to revise an initial decision (Alani et al., 2011). Knowledge gaps, such as whether PAAP poses a potential threat to general health (Aminoshariae et al., 2018; Khalighinejad et al., 2016; Sebring et al., 2022) also induce uncertainty.

Though uncertainty was revealed in this study, reluctance was a far more pronounced subcategory and exposed an abundance of negative emotions of different kinds among the informants. It was often a result of the informant being certain that an intervention was necessary but being overruled by the patient's autonomy. Dentists not only have to be skilled in the manual work of dentistry but also be pedagogical in explaining and educating their patients. When failing to reach through to the patient with important information, the informants suffered a long row of negative feelings including guilt and self‐reproach. In cases where the patient's self‐determination is diminished, as in the study on hospital patients, the dentists do not seemed to have to struggle with these emotions since no veto on the patients account really exist (Olsson et al., 2023). This paper revealed dissatisfaction, reluctance, frustration and a feeling of failure among dentists that had to agree to an inferior treatment plan which was in accordance with the patient's wishes but against the dentists recommendation (Röing & Holmström, 2014).

Few studies report other motives than clinical tooth‐related issues or respondents' situation (e.g. educational level, working experience, employment and geographical location) as influencing the decision‐making, but the patient's economy, preferences and lack of symptoms have also been acknowledged (Liew et al., 2022; Rawski et al., 2003; Taha et al., 2019). Our study elicited several additional factors that potentially influence the decision to treat or not to treat PAAP in RCFT including the dentists' own economy, emotions, convenience and ambition. To what degree such factors affected the decision‐making process, in this and other contexts, has to be further explored but offers a challenge as the subject might be perceived as sensitive.

There are few clearly stated normative theories for decision‐making about PAAP in RCFT. One is the interpretation of the dichotomizing of endodontic treatment results into ‘successes’ or ‘failures’, originally based on Strindberg (1956), this can also be used as a normative guide for clinical action; that is, if success—no further intervention, if failure—new intervention. In a critical examination of this ‘Strindberg concept’, it has been argued that the clinician has to follow a couple of so‐called prima facie principles; (1)—AP shall be treated or (2)—this can be overruled in accordance with the patient's right of self‐determination, or because of high costs, or risks versus benefit to the patient (Kvist, 2001, 2017). In the present study, in the context of management of actual patients in general dental practice, there were several examples of informants' arguments for not treating the tooth, in accordance with the second principle (Kvist, 2001, 2017).

The powerful protection of the patient's right to self‐determination, regulated in Swedish law (Socialdepartementet, 2019), is in accordance with internationally recognized encouragement of patient‐centred care, with a higher degree of patient involvement in the decision‐making process (Azarpazhooh et al., 2014; Chapple et al., 2003). Patient's wishes can however be manipulated through framing (Foster & Harrison, 2008; Kvist et al., 2023), and this can be used to nudge a patient towards a decision more convenient for the informant (Röing & Holmström, 2014).

Our study also revealed motives for withholding treatment that cannot be justified based on the second principle, which have not been previously reported. Some of these motives seemed to be emotionally driven. Several attitudes exposed in this study might be related to the stress, anxiety, discomfort, frustration and exhaustion which dentists associate with root canal treatment (Dahlström et al., 2017). Assumed negative consequences for the informant were revealed: fear of a bad reputation or losing the patient's trust in the case of an unsatisfactory outcome of an intervention. Patients have begun to behave more like market‐driven consumers (Röing & Holmström, 2014) and this could explain the informants' concerns of acquiring a negative reputation, as losing one or several clients could have a negative effect on the dentist's business. There are few studies of factors related to the dentist's personal preferences, wishes and characteristics in relation to decision‐making, but one interview study found that decision‐making was affected by the dentist's preferences and state of mind (Dawson et al., 2021).

The interviews revealed an inconsistency: that informants used a strategy of monitoring for some of the RCFT teeth with PAAP but on the other hand could regret taking a revealing X‐ray. The European Society of Endodontology recommend ‘that cases are monitored for a prolonged period with the review period extended if there is uncertainty about healing’ (Duncan et al., 2023). However, there are no recommendations for a strategy of watchful waiting for RCFT with confirmed PAAP.

A possible way to understand, interpret and further investigate practitioners' reluctance to treat PAAP is to examine their perception of different disease concepts. The starting point is conveniently the model discussed by Kvist and Hofmann (2023), where human maladies can be viewed from three perspectives; the professional and biological (‘disease’), the personal (‘illness’) and the social and cultural (‘sickness’). A hypothesis to investigate is, that although dentists are well aware that there is both infection and inflammation (‘disease’), the condition PAAP is not considered a ‘sickness’ in the sense that it should be treated as long as ‘illness’ (i.e. pain) does not occur concurrently.

In order to reduce uncertainty, an important task for clinical research is to map which and with what frequency serious negative effects occur in connection with the diagnosis of PAAP. And to additionally identify factors that can predict these events in detail. However, this study clearly demonstrates that GDPs decision‐making of PAAP in RCFT is not only a result of consideration about scientific evidence. Interpersonal relationships as well as the dentist's own preferences, and even emotions play an important role. Therefore, we suggest extended education of dental students and dentists on the matter of uncertainty and decision‐making. Furthermore, it seems of utmost importance to raise awareness that one self's convenience, concerns, ambitions, and emotions can interfere in the decision‐making process.

## CONCLUSION

The decision to refrain from further intervention, that is, ortho‐ or retrograde retreatment or extraction of an RCFT with PAAP was made with various degree of certainty, combined with compensatory strategies to support the decision. In addition to previously known patient and tooth related aspects, the GDP's, convenience, concerns, ambitions and emotions were elicited as important factors in the decision‐making process.

## AUTHOR CONTRIBUTIONS

Maria Granevik Lindström: Conceived the study and design, collected the material, contributed to analysis, writing and editing. Helena Fransson and Eva Wolf: Conceived the study and design, contributed to analysis, writing, editing and critical review. Victoria Dawson and Thomas Kvist: Contributed to analysis, writing, editing and critical review. All authors gave final approval and agreed to be accountable for all aspects of the work.

## FUNDING INFORMATION

The study was supported by grants from Uppsala County Council, Sweden.

## CONFLICT OF INTEREST STATEMENT

The authors state that there are no conflicts of interest related to this study.

## ETHICS STATEMENT

The Regional Ethical Review Board, Lund University, Lund, Sweden, determined that the study did not require a permit according to Swedish law, but issued an advisory statement approving the study, Dnr 2017/536. The research has been conducted in full accordance with ethical principles, including the World Medical Association Declaration of Helsinki (version 2013).

## Data Availability

Research data are not shared because of ethical restrictions and to avoid jeopardizing anonymity.
